# Comprehensive Characterization of Pyroptosis Patterns with Implications in Prognosis and Immunotherapy in Low-Grade Gliomas

**DOI:** 10.3389/fgene.2021.763807

**Published:** 2022-02-07

**Authors:** Zijian Zhou, Jinhong Wei, Bin Lu, Wenbo Jiang, Yue Bao, Luo Li, Weimin Wang

**Affiliations:** ^1^ Department of Neurosurgery, Qingdao Municipal Hospital, Qingdao University, Qingdao, China; ^2^ School of Basic Medical Sciences, Southwest Medical University, Luzhou, China

**Keywords:** low-grade glioma, pyroptosis, prognosis, tumor microenvironment, immunotherapy response

## Abstract

**Background:** Due to high heterogeneity and mortality of low-grade gliomas (LGGs), it is of great significance to find biomarkers for prognosis and immunotherapy. Pyroptosis is emerging as an attractive target in cancer research for its effect on tumor immune microenvironment (TIME). However, the investigation of pyroptosis in LGGs is insufficient.

**Methods:** LGG samples from TCGA and CGGA database were classified into two pyroptosis patterns based on the expression profiles of 52 PRGs using consensus clustering. A prognostic model was constructed by using the LASSO-COX method. ESTIMATE algorithm and single sample gene set enrichment analysis (ssGSEA) were used to characterize the TIME. Based on the differentially expressed genes between two pyroptosis patterns, favorable and unfavorable pyroptosis gene signatures were determined. Pyroptosis score scheme was constructed to quantify the pyroptosis patterns through gene set variation analysis (GSVA) method. Two external datasets and immunotherapy cohort from CGGA and GEO database were used to validate the predictive value of the pyroptosis score. The Human Protein Atlas website and Western blotting were utilized to confirm the expression of the selected genes in the prognostic model in LGGs.

**Results:** Distinct overall survival and immune checkpoint blockage therapeutic responses were identified between two pyroptosis patterns. A low pyroptosis score in LGG patients implies higher overall survival, poor immune cell infiltration, and better response to immunotherapy of immune checkpoint blockage.

**Conclusion:** Our findings provided a foundation for future research targeting pyroptosis and opened a new sight to explore the prognosis and immunotherapy from the angle of pyroptosis in LGGs.

## Introduction

Low-grade gliomas (LGGs) represent a group of common malignant tumors in the central nervous system especially for younger patients, mainly composed of grade II-III gliomas including astrocytoma, oligoastrocytoma, and oligodendroglioma according to the World Health Organization classification system, and differ with high-grade glioma (glioblastoma, GBM) in biological and clinicopathological characteristics (Cancer Genome Atlas Research et al., 2015; Hoshide and Jandial, 2016; Ostrom et al., 2018; Sidaway, 2020). Due to the high heterogeneity of LGGs, the traditional classification is not satisfactory for predicting the prognosis even for patients with the same diagnosis. Considering the limited therapeutic effects and related complications, conventional strategies including surgical resection, chemotherapy, and radiotherapy cannot reverse the poor prognosis of LGG patients (Duffau and Taillandier, 2014; Darlix et al., 2018). As a novel therapeutic strategy, immunotherapy has been extensively investigated in more and more cancers and durable responses to immune checkpoint blockage (ICB) treatment in many other forms of cancers have drawn increasing attention in gliomas (McGranahan et al., 2019). However, not all patients could get efficient responses to immunotherapy due to lack in precise selection with predictive biomarkers (Chiocca et al., 2019).

Pyroptosis, recently identified as gasdermins (GSDMs)-mediated programmed cell death, is characterized by lytic, featuring cell swelling, and large bubbles blowing from the plasma membrane (Jorgensen and Miao, 2015). Previous study revealed that low expression level of gasdermin D (*GSDMD*) correlated with a favorable prognosis in non-small-cell lung cancer (Gao et al., 2018). The expression of *GSDMA* in human gastric cancers was suppressed and considered as a tumor suppressor gene (Saeki et al., 2000). For its immune defense function, pyroptosis is recognized as a general innate immune effector mechanism and involved in the regulation of tumor-immune microenvironment (TIME) (Jorgensen and Miao, 2015; Erkes et al., 2020). The infiltrating immune cells in TIME are closely related to the prognosis for LGG patients (Hottinger et al., 2016). CD8^+^ T cell-dependent antitumor immunity was activated by the induction of pyroptosis in melanoma. A number of studies demonstrated that pyroptosis plays a crucial role in antitumor immunity and the induction of pyroptosis has been emerging as a promising therapeutic strategy in cancers (Johnson et al., 2018; Fan et al., 2019; Erkes et al., 2020). To date, there are rare studies focusing on the pyroptosis related molecular patterns with implications in prognosis and immunotherapy in LGGs. Considering the effect of pyroptosis on TIME and antitumor immunity, identification of the pyroptosis patterns, and related gene signatures displays an indispensable advantage in predicting prognosis and immunotherapy response in LGGs.

In this study, we identified two pyroptosis related molecular patterns with distinct prognosis and TIME, based on which we developed a pyroptosis scoring scheme with appealing implications in predicting the prognosis and immune therapy in LGGs. Additionally, a prognostic model was constructed based on 52 pyroptosis related genes (PRGs) to confirm the prognostic values of PRGs. Our findings provided a foundation for future research targeting pyroptosis and opened a new sight to explore the prognosis and immunotherapy from the angle of pyroptosis in LGGs.

## Materials and methods

### Multiomic Data Acquisition

The RNA sequencing (RNA-seq) data consisting of 508 LGGs samples and the corresponding clinical information were obtained from The Cancer Genome Atlas (TCGA, http://cancergenome.nih.gov/) database. The RNA annotation file of Genome Reference Consortium Human Build 38 (GRCh38) was downloaded from the Ensembl website (http://asia.ensembl.org/) for annotation of RNA-seq data. The data set (DataSet ID: mRNA-array_301) including 159 LGG samples, were downloaded from the Chinese Glioma Genome Atlas (CGGA, http://cgga.org.cn/index.jsp) (Fang et al., 2017; Wang et al., 2017). We collected a total of 52 PRGs through scanning the associated literatures (Shi et al., 2017; Wang et al., 2020a; Wang et al., 2020b; Broz et al., 2020; Zhou et al., 2020; Tan et al., 2021). Based on the transcriptomic data, the correlation among the PRGs was identified through co-expression analysis in which the cut off of the correlation coefficient was set at 0.7. The somatic mutation data (MAF format) of 503 LGG samples based on the whole exome sequencing platform were also downloaded from the TCGA database. The mutation types and frequencies of PRGs were analyzed and visualized in oncoplot by using the maftools package in R (Mayakonda et al., 2018). Tumor mutation burden (TMB), which was closely related to immune microenvironment, was defined by the cumulative nonsynonymous mutations in per million bases in coding regions. The copy number variation (CNV) data (n = 527) for LGG samples were downloaded from the University of California Santa Cruz (UCSC) Xena browser (https://xena.ucsc.edu/) (Goldman et al., 2020). The variation frequencies for the gain/loss alterations of PRGs were analyzed and visualized in a bar plot. The chromosomal positions of the crucial PRGs were displayed by Circos plot using the RCircos package in R (Zhang et al., 2013). Perl software (version 5.32.1.1) and R software (version 4.1.0) were involved in the processing of the data.

### Identification of Pyroptosis Related Molecular Patterns Through Consensus Clustering Analysis

The RNA-seq data of LGG samples from the TCGA database were transformed to transcripts per million (TPM) values and log2-scale transferred and subsequently merged with transcriptomic data of LGG samples from the CGGA database (DataSet ID: mRNA-array_301). The transcriptomic data were normalized and corrected batch effect for further analysis by using the sva package in R (Leek et al., 2012). The unsupervised consensus clustering method was utilized to determine the pyroptosis related molecular patterns based on the expression profiles of PRGs in LGGs by using ConsesusClusterPlus package in R software (Wilkerson and Hayes, 2010). The clustering procedure, with 50 iterations, was performed based on 80% of the samples in the dataset in each iteration. The optimal number for the classification of LGG patients was determined by comprehensive analysis of the consensus matrix heatmap and the relative change in the area under the cumulative distribution function (CDF) curves of consensus scores. Principal component analysis (PCA) was employed to examine the subtype assignment. Kaplan-Meier analysis was used to compare the overall survival between different pyroptosis patterns in which the log-rank test was used for statistical analysis.

### Identification of the Tumor Immune Microenvironment and Immunogenomic Features

The scores of tumor-infiltrating immune cells and the associated immune functions for each LGG sample were estimated through single sample gene set enrichment analysis (ssGSEA) based on the expression levels of marker genes in the input data set by using GSEABase and GSVA R packages (Hänzelmann et al., 2013). The comparisons of the infiltrating immune cells and immune functions were conducted and visualized in box plots. ESTIMATE algorithm was used to evaluate the TIME of each sample (Yoshihara et al., 2013). The response to immune checkpoint blockage (ICB) was estimated by Tumor Immune Dysfunction and Exclusion (TIDE; http://tide.dfci.harvard.edu/login/) website.

### Gene Set Variation Analysis

GSVA was applied to explore the underlying molecular mechanisms for different pyroptosis patterns by using the GSVA package in R (Hänzelmann et al., 2013). The significantly enriched gene ontology (GO) molecular function terms and Kyoto Encyclopedia of Genes and Genomes (KEGG) pathways between the two pyroptosis patterns were analyzed by using the limma package in R (Smyth et al., 2005). |log2 fold change (FC)| > 0.1 and false discovery rate (FDR) adjusted *p* values < 0.05 were considered statistically significant. “c5.go.mf.v7.4.symbols” and “c2.cp.kegg.v7.4.symbols” downloaded from GSEA database were selected as the reference files.

### Construction of the Prognostic Model Based on PRGs

First, the RNA-seq data of LGG samples from the TCGA database, which were treated as the training cohort, were TPM and log2-scale transferred as described above. The transcriptomic data of LGG samples from the CGGA database (DataSet ID: mRNA-array_301) were treated as the validation cohort. The transcriptomic data involved in the validation cohort and training cohort were normalized and corrected batch effect for further analysis. The expression profiles of 52 PRGs in the two cohorts were obtained. Univariate cox regression analysis was utilized to screen out the PRGs with prognostic values by using survival package in R, in which *p* < 0.05 was considered as statistically significant. Afterward, the least absolute shrinkage and selection operator (LASSO) regression algorithm was employed to construct the prognostic model based on the expression profiles of prognostic PRGs. The receiver operating characteristic (ROC) curves were used to evaluate the predictive efficacy of the prognostic model in which survival, glmnet, survminer, and timeROC packages in R were employed. The risk score for each patient was calculated following the formula: risk score = $\overset{n}{\underset{i=1}{\sum }}coef\text{PRG}i\text{*}EXP\text{ PRG}i$ in which the  coefPRGi means the coefficient for the i th PRG, and the EXP PRGi represents the expression level of the i th PRG in the prognostic model. Univariate and multivariate cox regression analysis were conducted to explore the prognostic value of the risk score. Patients were divided into high-risk group and low-risk group according to the cut off of the median risk score. PCA and the t-distributed stochastic neighbor embedding (tSNE) algorithm were utilized to evaluate the assignment of the subgroups.

Nomogram combing risk score and clinicopathological factors were introduced to fulfill the prognostic model by using “rms” and “regplot” R package. Predictions for survival at the time of 1, 3, and 5 years were accomplished. Calibration curves were carried out to evaluate the accuracy of the nomogram.

### Pyroptosis Scoring via GSVA

Differentially expressed genes (DEGs) between the two pyroptosis patterns were identified with |log2 FC| > 0 and FDR < 0.001 by limma package in R. Univariate cox regression analysis was carried out to screen out DEGs with prognostic values by using survival package in R, in which *p* < 0.05 was considered as statistically significant. Based on the expression profiles of DEGs with prognostic values, which were considered as pyroptosis gene signatures, LGG patients were separated into two gene clusters by unsupervised consensus clustering analysis.

The pyroptosis gene signatures which had favorable and unfavorable correlations with the prognosis of LGG patients were respectively defined as favorable and unfavorable pyroptosis related gene sets. GSVA method was utilized to evaluate the enrichment score (GSVA score) of the two pyroptosis related gene sets (unfavorable and favorable gene sets) for each LGG sample by using the GSVA package in R software (Hänzelmann et al., 2013). GSVA is a popular method for scoring individual samples based on molecular characteristics or gene sets and a gene expression dataset. GSVA represents a x method that estimates the variation of a specific function activity over a sample population in an unsupervised manner. According to the method described by Hänzelmann et al., GSVA score of the unfavorable and favorable gene sets for each sample was calculated. The pyroptosis score for each sample was calculated as follows: pyroptosis score=GSVAscore2−GSVAscore1 , where GSVAscore2 represented the GSVA score of the unfavorable pyroptosis related gene set and GSVAscore1 represented the GSVA score of favorable pyroptosis related gene set. Survminer package in R was used to determine the optimal cut-off value of the pyroptosis score for each subgroup.

### Validation of Pyroptosis Score for Predicting the Prognosis and Immunotherapy Response in External Data Sets

LGG samples involved in four data sets (GSE4271, GSE4412, GSE43378, GSE84010) obtained from the Gene Expression Omnibus (GEO, https://www.ncbi.nlm.nih. gov/geo/) database were merged into one data set to verify the prognostic value of pyroptosis score in LGGs. The RNA-seq data of LGG samples (DataSet ID: mRNAseq_325) from CGGA database were defined as another validation cohort (Bao et al., 2014; Zhao et al., 2017). Furthermore, the GSE78220 data set from the GEO database was applied to validate the predictive value of the pyroptosis score in anti-PD1 immunotherapy response.

### Validation of the Selected Genes in the Prognostic Model at Protein Level

We randomly selected four genes (CASP3, CASP8, GSDMD, PLCG1) from the unfavorable gene set and identified the differential expression of the four unfavorable pyroptosis genes at the protein level between normal brain tissues and LGG tissues on the Human Protein Atlas website (https://www.proteinatlas.org/) (Colwill et al., 2011).

Western blotting was applied to verify the differential expression of the four unfavorable pyroptosis genes. Brain tissues obtained from patients with epilepsy who received temporal lobe resection were treated as control groups. Astrocytoma tissues which were histologically diagnosed as grade II (G2) gliomas were obtained from LGG patients who received tumor resection. Oligodendroglioma tissues which were histologically diagnosed as grade III (G3) gliomas were obtained from LGG patients who received tumor resection.

The collected frozen tissues were homogenized and lysed in ice-cold lysis solution consisting of 1.0 mmol/L PMSF (phenylmethylsulfonyl fluoride), 2.5 mmol/L EDTA, 1 mmol/L EGTA, 15 mmol/L Tris (pH 7.6), 2.5 mg/ml aprotinin, 1.25 mg/ml pepstatin A, 10 mg/ml leupeptin, 1 mmol/L dithiothreitol, 2.0 mmol/L Na4P2O7, 1.0 mmol/L MgCl2, 0.1 mmol/L Na3VO4, 50 mmol/L NaF, and 250 mmol/L sucrose. The homogenized suspension was centrifuged at 1000 g for 15 min at 4°C and the protein content in the supernatant was examined and regulated to equal level by using Bio-Rad protein assay kit. Loading buffer including sodium dodecylsulfate (SDS) was homogenized with the protein suspension and then boiled for 5 min at 100°C. The same amounts of samples were added and electrophoresed on 10% SDS gels at 100 V for 50 min. Afterward, the protein on the gels was transferred to PVDF membranes at 60 V for 45 min. The membranes were immersed in 3% bovine serum albumin for 45 min and then incubated in antibody solutions containing anti-caspase3 (1:1000), anti-caspase8 (1:1000), anti-caspase4 (1:1000), anti-PLCG1 (1:1000), anti-TP63 (1:1000), anti-caspase9 (1:1000), and anti-β-actin (1:1000) antibodies, respectively at 4°C for 12 h. Subsequently, the membranes were rinsed in TBS (Tris buffered saline) for 30 min and incubated with horseradish peroxidase-conjugated goat anti-rabbit or anti-mouse IgG (1:2000) solutions for 2 h. Then the blot membranes were rinsed and visualized on Kodak X-omat LS films with enhanced chemiluminescence.

### Statistical Analysis

The Wilcoxon test was implemented to compare two groups. Kruskal-Wallis tests were used to compare the differences between multiple groups. The distribution of categorical variables between subgroups was compared by Chi-square tests. The Student’s t-test was utilized to compare the continuous data between two groups. Two-sided *p* < 0.05 was considered statistically significant.

## Results

### Genomic Variations of the PRGs

**FIGURE 1:**
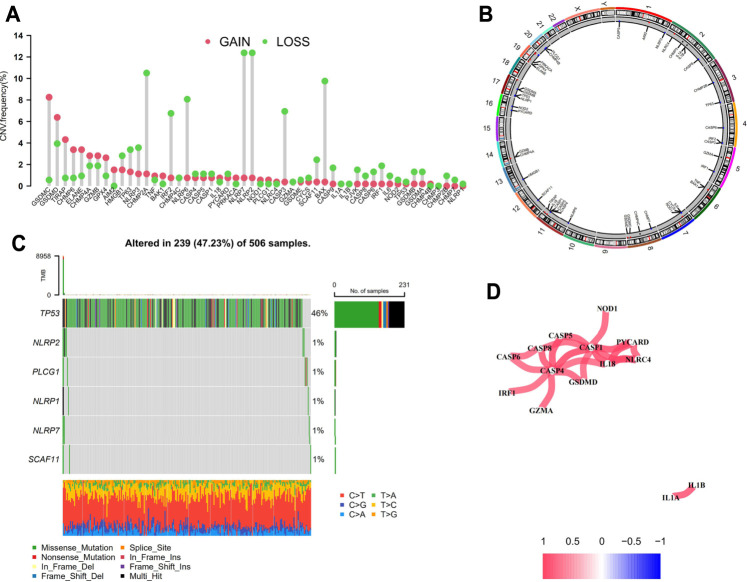
Genomic variations of the PRGs in LGGs. **(A)** Bar plot displaying the copy number variation frequencies of gain/loss alterations of the PRGs. **(B)** Circos plot showing the positions of the PRGs in chromosomes in which red dots indicate amplifications, blue dots indicate deletions, and black dots represent no significant variations. **(C)** Oncoplot of six mutated PRGs in LGG samples. **(D)** Co-expression analysis revealed the correlation among the 52 PRGs. PRGs, pyroptosis related genes; LGGs, low-grade gliomas.

The CNVs including gain and loss alterations of the 52 PRGs in LGG samples are shown in Figure 1A. As the pyroptosis executioner, *GSDMD* displayed significant amplifications of copy number (18), while *GSDMA* and *GSDMB* showed deletions. The corresponding chromosomal locations of the PRGs were displayed in Figure 1B. Most of the PRGs showed low or no mutations in LGGs (Figure 1C). Co-expression analysis noted that *CASP1* and *CASP4* positively correlated with most of the PRGs (Figure 1D). Moreover, the positive correlation across *GSDMD*, *CASP1*, and *CASP4* suggested the underlying molecular interactions.

### Pyroptosis Related Molecular Patterns with Distinct Prognosis and TIME Features

**FIGURE 2:**
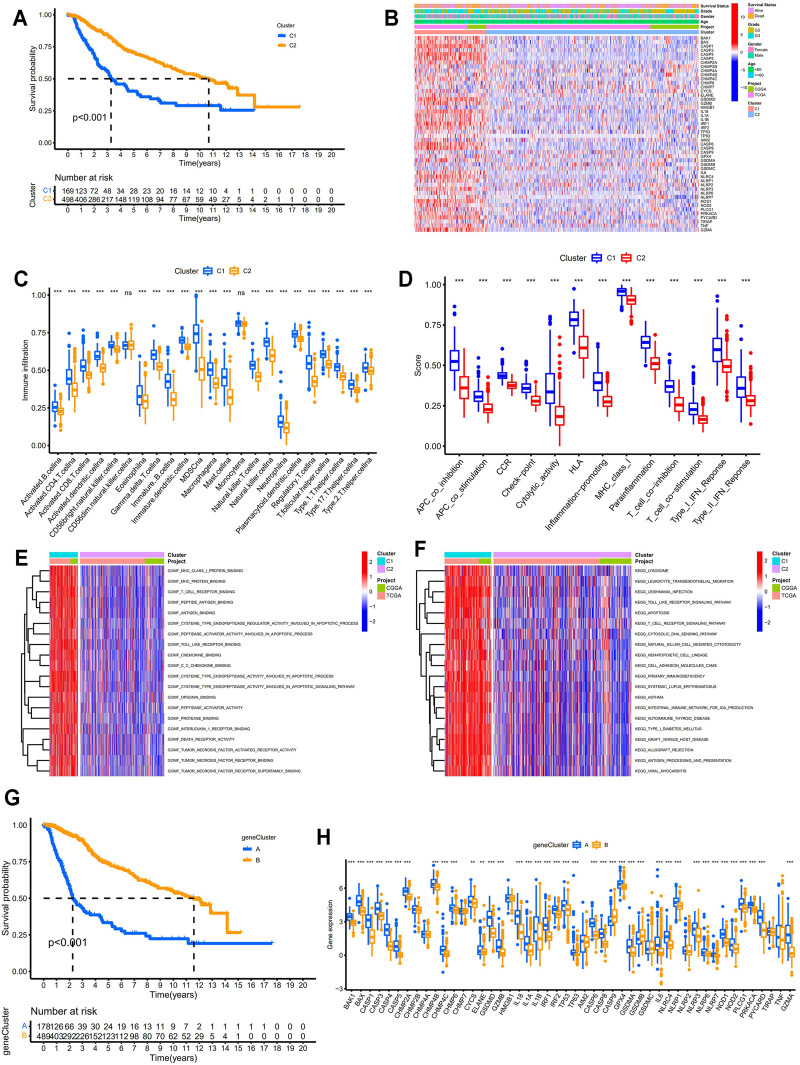
Identification of pyroptosis-related molecular patterns and gene clusters in LGGs. **(A)** Kaplan–Meier survival analysis between two pyroptosis patterns. **(B)** Heatmap displaying the differential expression patterns of the PRGs between two pyroptosis patterns. **(C)** Differential analysis of the abundance of infiltrating immune cells between two pyroptosis patterns. **(D)** Differential analysis of the immune responses between two pyroptosis patterns. **(E,F)** Functional enrichment analysis between two pyroptosis patterns. **(G)** Kaplan–Meier survival analysis between two pyroptosis-related gene clusters. **(H)** Differential analysis of the expression levels of PRGs between two pyroptosis-related gene clusters. * means *p* < 0.05, ** means *p* < 0.01, and ***means *p* < 0.001. PRGs, pyroptosis related genes; LGGs, low-grade gliomas.

Based on the expression profiles of 52 PRGs in LGG samples from the TCGA database and the CGGA database (DataSet ID: mRNA-array_301), LGG samples were divided into two molecular patterns. As shown in Supplementary Figure S1A, two samples were more likely to be grouped into the same cluster when there was a higher consensus score between them in the consensus matrix heatmap. We found extremely high consensus scores between samples in the same cluster and low consensus scores between samples in different clusters when samples were classified into two clusters (k = 2). In addition, no appreciable increase (the relative change = 0.4) was observed in the area under the CDF curve when the number of clusters was determined to be two (k = 2). Hence, LGG patients were categorized into two clusters, which were termed as two pyroptosis related molecular patterns. PCA of the expression profiles of PRGs from LGG samples confirmed the two pyroptosis related molecular patterns, suggesting that we could distinguish two clusters based on the expression profiles of PRGs in LGGs (Supplementary Figure S1B). Patients in C1 presented significantly lower overall survival compared with those in C2 (*p* < 0.001; Figure 2A). Furthermore, the expression levels of most of the PRGs were higher in C1 and the clinicopathological characteristics differed between the two pyroptosis patterns (Figure 2B). As shown in Figure 2C, almost all the immune cells significantly infiltrated in the TIME of C1 (*p* < 0.001). All the immune response involved in this study tended to be more active in C1 especially cytolytic activity (*p* < 0.001; Figure 2D). Molecular functions such as peptidase activator activity involved in apoptosis process, death receptor activity, and immune related functions significantly enriched in C1 (Figure 2E). KEGG pathway enrichment analysis suggested more apoptosis and immune related pathways were active in C1 (Figure 2F). All these findings indicated the pyroptosis related molecular biological processes were more active in C1. Based on the expression profiles of DEGs between the two pyroptosis related molecular patterns, LGG patients were further classified into two gene clusters (Supplementary Figure S1C). PCA revealed that the two subgroups can be well distinguished (Supplementary Figure S1D). The overall survival for LGG patients in gene cluster A was significantly lower than those in gene cluster B (*p* < 0.001; Figure 2G). The expression levels of the PRGs were significantly higher in gene cluster A which seemed consistent with the classification based on the pyroptosis patterns.

### Construction of the Prognostic Model Based on PRGs

**FIGURE 3:**
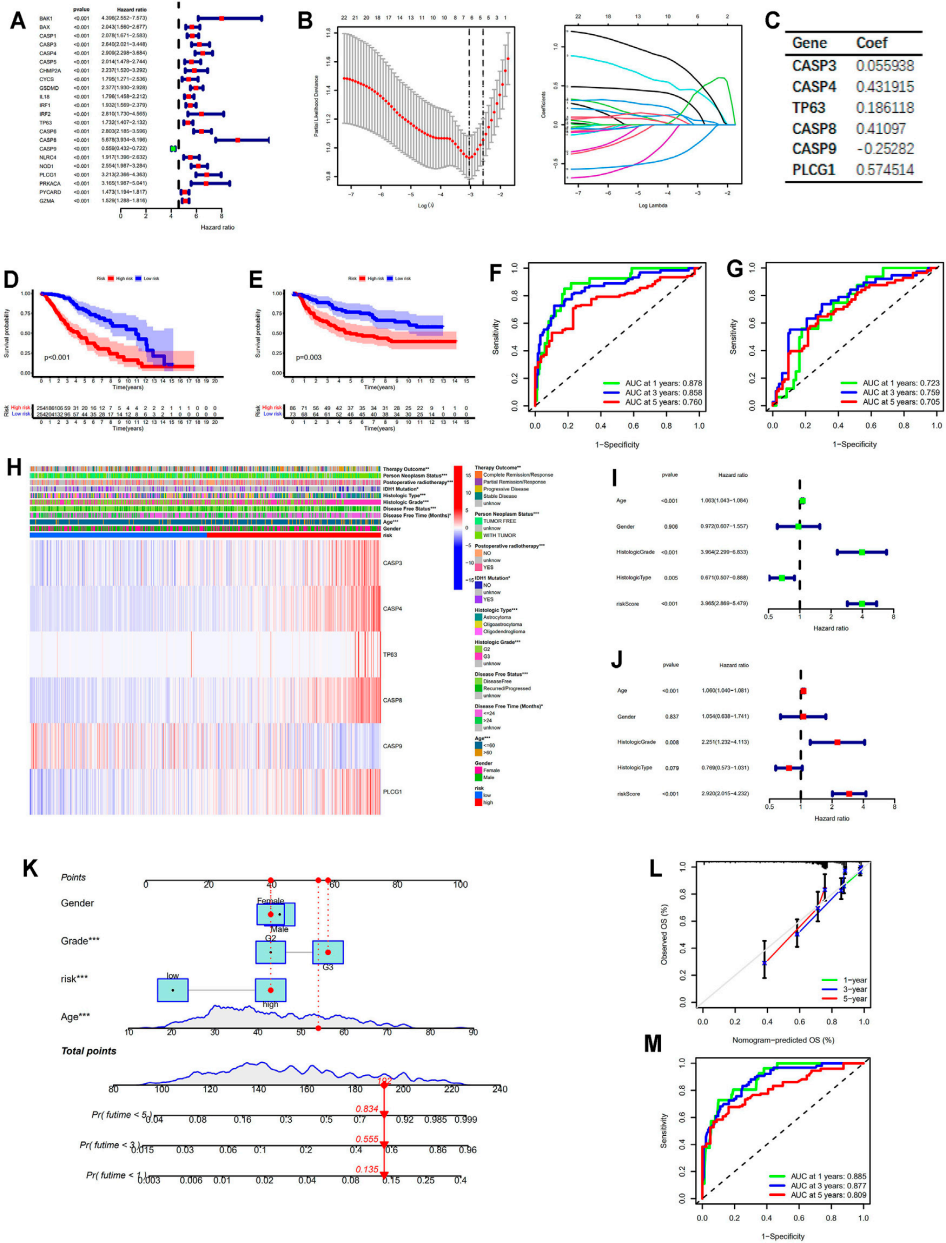
Construction of the prognostic model based on PRGs. **(A)** Forest plot showing the prognostic PRGs based on univariate cox regression analysis. **(B)** The optimal parameter (lambda) selection and coefficient profile plot against the log (lambda) sequence in the LASSO model. **(C)** The critical PRGs with the corresponding coefficients in the prognostic model. **(D,E)** Kaplan–Meier survival analysis between the low- and high-risk groups in the training and validation cohort, respectively. **(F,G)** ROC curves for the prognostic model in the training and validation cohort, respectively. **(H)** Heatmap displaying the expression patterns of six PRGs involved in the model in the training cohort. **(I,J)** Forest plots showing the results of univariate and multivariate cox regression analysis for the risk score in the training cohort. **(K)** Nomogram integrating risk and multiple clinicopathological features in the training cohort. **(L)** Calibration curves of the nomogram in the training cohort. **(M)** ROC curves for the nomogram in the training cohort. * means *p* < 0.05, ** means *p* < 0.01, and ***means *p* < 0.001. PRGs, pyroptosis related genes; LASSO, least absolute shrinkage and selection operator; ROC, receiver operating characteristic.

Based on the expression levels of 22 prognostic PRGs which were screened out through univariate cox regression analysis (*p* < 0.001; Figure 3A), the LASSO regression algorithm was used to construct the prognostic model (Figure 3B). Six PRGs involved in the prognostic model were listed in Figure 3C. The risk score for each patient was calculated following the formula described above. Patients were classified into high- and low-risk group with the cut off of the median risk score in the training and validation cohort, respectively. PCA and t-SNE verified the assignment of the subgroups (Supplementary Figures S2A and S2B). Patients in the low-risk group exhibited significantly longer survival time either in the training or the validation cohort (*p* < 0.001 and *p* = 0.003, respectively, Figures 3D,E). The ROC curves indicated that the risk score based on the prognostic model can be a reliable predictor for prognosis either in the training cohort (AUC for 1, 3, 5 years: 0.878, 0.858, 0.760, respectively, Figure 3F) or validation cohort (AUC for 1, 3, 5 years: 0.723, 0.759, 0.705, respectively, Figure 3G). The heatmap in Figure 3H displayed the expression patterns of the six PRGs involved in the model and the correlation between the risk score and the multiple clinicopathological features. The univariate and multivariate cox regression analysis confirmed the prognostic values of the risk score in the training (*p* < 0.001; Figures 3I,J) and validation cohort (*p* < 0.001; Supplementary Figures S2C and S2D). Nomogram integrating risk and multiple clinicopathological features was established for clinical practice in the training (Figure 3K) and validation cohort (Supplementary Figure S2E). Calibration curves for predicting 1 year, 3 years, and 5 years overall survival were close to the actual observed values in the training (Figure 3L) and validation cohort (Supplementary Figure S2F). Additionally, the values for AUC of the nomogram for predicting 1 year, 3 years, and 5 years overall survival were 0.885, 0.877, 0.809, respectively, in the training cohort (Figure 3M) and 0.831, 0.802, 0.795, respectively, in the validation cohort (Supplementary Figure S2G). All these results revealed the powerful performance of the nomogram in predicting prognosis in LGG patients.

### Pyroptosis Score Served as a Powerful Prognostic Factor

**FIGURE 4:**
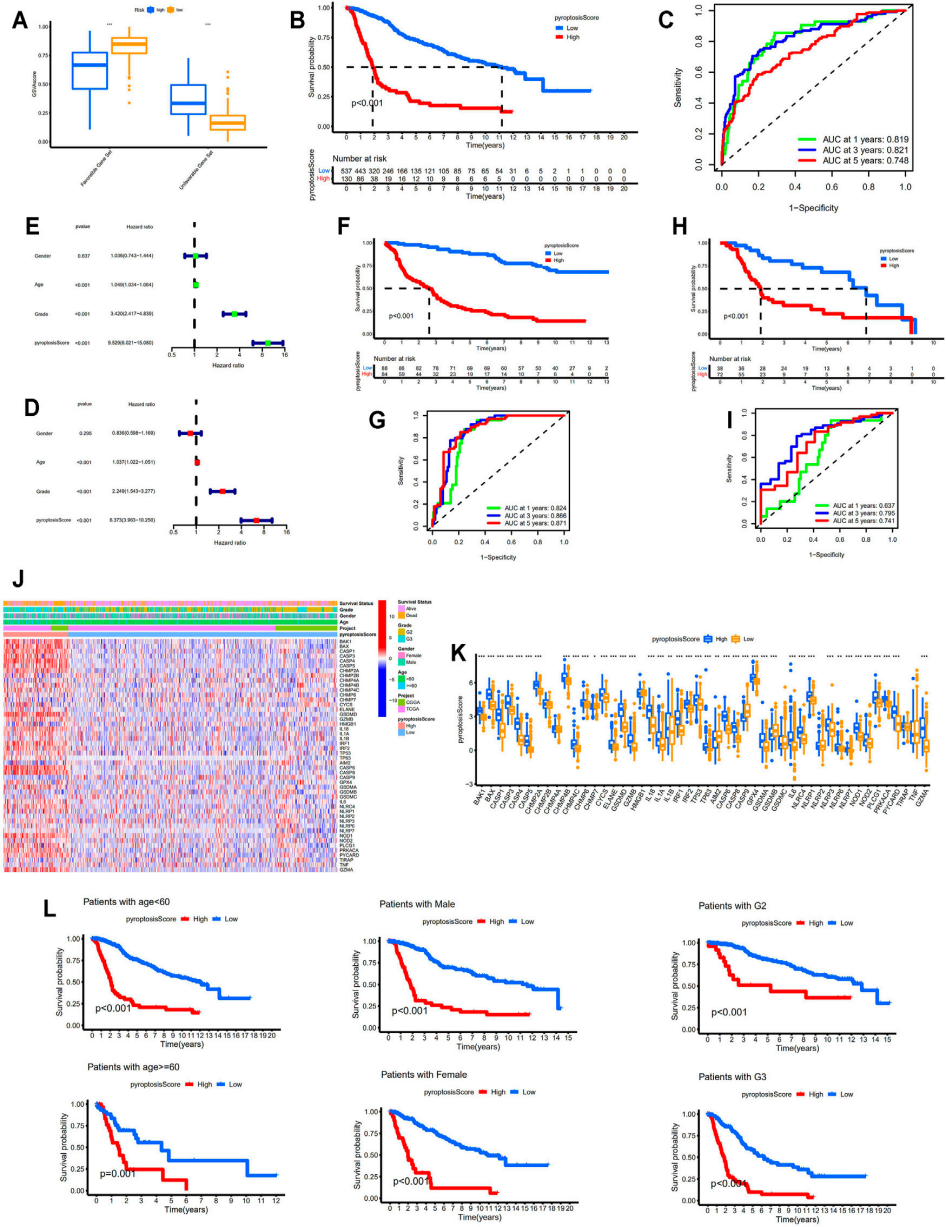
Identification of the correlation between pyroptosis score and prognosis. **(A)** Comparisons of the GSVA scores for favorable and unfavorable gene set between the low- and high-risk groups. **(B)** Kaplan–Meier survival analysis between the low- and high-pyroptosis score groups. **(C)** ROC curves for pyroptosis score. **(D,E)** Forest plots showing the results of univariate and multivariate cox regression analysis for pyroptosis score. **(F,G)** Verification of the above results in the validation cohort from the CGGA database (DataSet ID: mRNAseq_325). **(H,I)** Verification of the above results in the validation cohort from the GEO database. **(J,K)** Identification of the differential expression patterns of the PRGs between the low- and high-pyroptosis score groups. **(L)** Kaplan–Meier survival analysis between the low- and high-pyroptosis score subgroups with different clinicopathological features. * means *p* < 0.05, ** means *p* < 0.01, and ***means *p* < 0.001. GSVA, gene set variation analysis; ROC, receiver operating characteristic; PRGs, pyroptosis-related genes.

The GSVA scores for favorable pyroptosis gene set in the low-risk group were significantly higher than those in the high-risk group, while the GSVA scores for unfavorable pyroptosis gene set in the low-risk group were significantly lower compared with the high-risk group implying that the GSVA scores for the pyroptosis gene signatures closely correlated with prognosis (*p* < 0.001; Figure 4A). Based on the GSVA scores for the pyroptosis gene signatures, pyroptosis scores were calculated according to the mentioned method. The clinical data for LGG samples with low and high pyroptosis scores are demonstrated in Tables 1 and 2. Kaplan–Meier survival analysis revealed that LGG patients with higher pyroptosis scores presented pessimistic prognosis (*p* < 0.001; Figure 4B). The ROC curves with AUC for 0.819, 0.821, and 0.748 (for predicting 1, 3, and 5 years overall survival, respectively) verified the accuracy of the pyroptosis score for predicting prognosis (Figure 4C). The univariate cox analysis indicated that the pyroptosis score significantly correlated with survival (*p* < 0.001; Figure 4D). The multivariate cox analysis suggested that the pyroptosis score can be an independent factor for predicting prognosis in LGGs (*p* < 0.001; Figure 4E). Two validation cohorts from the CGGA database (Figures 4F,G) and the GEO database (Figures 4H,I), respectively, verified the predictive values of the pyroptosis score. The corresponding clinical information for two validation cohorts was listed in Supplementary Tables S1 and S2. The expression levels of most of the PRGs were significantly higher in the high-pyroptosis score group (Figures 4J,K). LGG patients in the high-pyroptosis score subgroups with distinct clinicopathological features tended to get worse prognosis compared with the low-pyroptosis score subgroups (all *p* ≤ 0.001; Figure 4L), which was consistent with the above results.

**TABLE 1:** Clinical features of patients with low-grade gliomas in TCGA database.

Covariates		Total	High-pyroptosis score	Low-pyroptosis score
Gender	Female	226 (44.49%)	42 (44.21%)	184 (44.55%)
	Male	282 (55.51%)	53 (55.79%)	229 (55.45%)
Age	<60	439 (86.42%)	68 (71.58%)	371 (89.83%)
	≥60	69 (13.58%)	27 (28.42%)	42 (10.17%)
Grade	G2	246 (48.43%)	14 (14.74%)	232 (56.17%)
	G3	261 (51.38%)	81 (85.26%)	180 (43.58%)
	Unknown	1 (0.2%)	0 (0%)	1 (0.24%)
Histologic type	Astrocytoma	192 (37.8%)	66 (69.47%)	126 (30.51%)
	Oligoastrocytoma	128 (25.2%)	15 (15.79%)	113 (27.36%)
	Oligodendroglioma	188 (37.01%)	14 (14.74%)	174 (42.13%)
IDH1 mutation	YES	91 (17.91%)	8 (8.42%)	83 (20.1%)
	NO	34 (6.69%)	19 (20%)	15 (3.63%)
	Unknown	383 (75.39%)	68 (71.58%)	315 (76.27%)

**TABLE 2:** Clinical features of patients with low-grade gliomas in CGGA database (DataSet ID: mRNA-array_301).

Covariates		Total	High-pyroptosis score	Low-pyroptosis score
Type	Primary	143 (89.94%)	28 (80%)	115 (92.74%)
	Recurrent	16 (10.06%)	7 (20%)	9 (7.26%)
Grade	G2	106 (66.67%)	12 (34.29%)	94 (75.81%)
	G3	53 (33.33%)	23 (65.71%)	30 (24.19%)
Gender	Female	69 (43.4%)	12 (34.29%)	57 (45.97%)
	Male	90 (56.6%)	23 (65.71%)	67 (54.03%)
Age	<60	146 (91.82%)	28 (80%)	118 (95.16%)
	≥60	11 (6.92%)	6 (17.14%)	5 (4.03%)
	Unknown	2 (1.26%)	1 (2.86%)	1 (0.81%)
Radio status	Treated	136 (85.53%)	29 (82.86%)	107 (86.29%)
	Untreated	21 (13.21%)	6 (17.14%)	15 (12.1%)
	Unknown	2 (1.26%)	0 (0%)	2 (1.61%)
Chemo status (TMZ)	Treated	69 (43.4%)	23 (65.71%)	46 (37.1%)
	Untreated	85 (53.46%)	12 (34.29%)	73 (58.87%)
	Unknown	5 (3.14%)	0 (0%)	5 (4.03%)
IDH status	Mutant	105 (66.04%)	14 (40%)	91 (73.39%)
	Wildtype	53 (33.33%)	20 (57.14%)	33 (26.61%)
	Unknown	1 (0.63%)	1 (2.86%)	0 (0%)
1p19q codeletion status	Codel	16 (10.06%)	0 (0%)	16 (12.9%)
	Non-codel	34 (21.38%)	10 (28.57%)	24 (19.35%)
	Unknown	109 (68.55%)	25 (71.43%)	84 (67.74%)
MGMTp methylation status	Methylated	43 (27.04%)	10 (28.57%)	33 (26.61%)
	Un-methylated	107 (67.3%)	23 (65.71%)	84 (67.74%)
	Unknown	9 (5.66%)	2 (5.71%)	7 (5.65%)

### Pyroptosis Score Served as a Predictor for TIME and Immunotherapy Response

**FIGURE 5:**
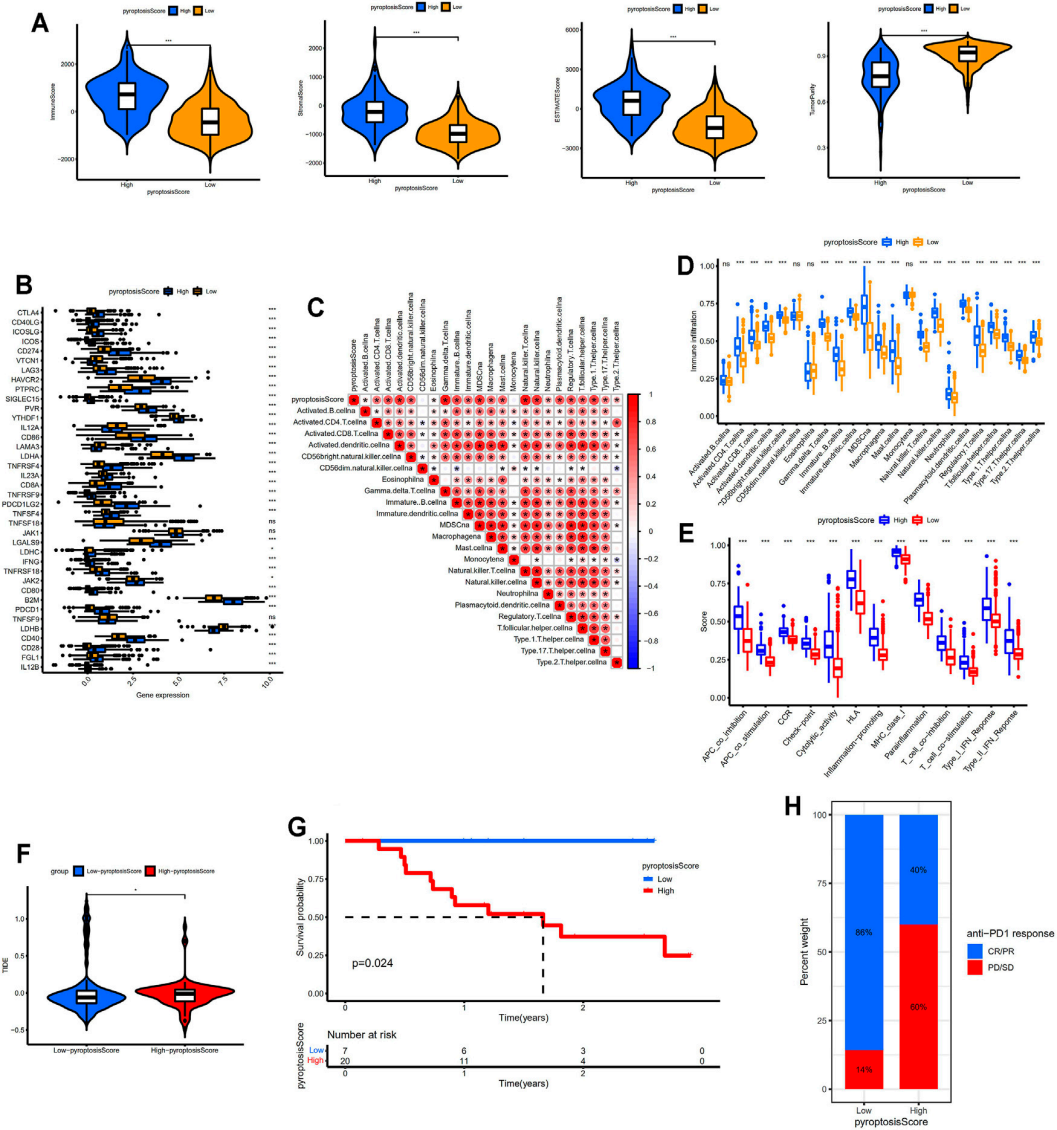
Identification of distinct TIME and immunotherapy response between different pyroptosis score groups. **(A)** Comparisons of immune, stromal, ESTIMATE scores, and tumor purity between two groups. **(B)** Comparisons of the expression levels of immune check points between two groups. **(C)** Correlation between the pyroptosis score and infiltrating immune cells. **(D)** Comparisons of the abundance of infiltrating immune cells between two groups. **(E)** Comparisons of the immune functions between two groups. **(F)** Comparison of TIDE scores between two groups. **(G)** Kaplan–Meier survival analysis between the low- and high-pyroptosis score groups in GSE78220 data set. **(H)** Bar plot showing different responses to anti-PD1 immunotherapy between two groups. * means *p* < 0.05, ** means *p* < 0.01, and ***means *p* < 0.001. TIME, tumor immune microenvironment; TIDE, Tumor Immune Dysfunction and Exclusion; CR/PR, complete response/partial response; PD, progressed disease; SD, stable disease.

The immune, stromal, and ESTIMATE scores were distinctly higher in the high-pyroptosis score group indicating more immune and stromal cells in TIME. On the contrary, lower tumor purities were determined in the high-pyroptosis score group compared with the low-pyroptosis score group (*p* < 0.001; Figure 5A). The expression levels of immune check points including *PDCD1*, *CD274*, *PDCD1LG2*, *CTLA4*, *CD80*, and *CD86* were significantly higher in the high-pyroptosis score group (*p* < 0.001, Figure 5B). As shown in Figure 5C, the pyroptosis score positively correlated with most of the infiltrating immune cells in TIME especially activated dendritic cells, Gamma delta T cells, natural killer cells, and type 1 T helper cells. The high-pyroptosis score group presented significantly higher scores of infiltrated immune cells than the low-pyroptosis score group (Figure 5D). Patients in the high-pyroptosis score group exhibited higher scores of immune functions such as checkpoint, cytolytic activity, and para-inflammation (Figure 5E). TIDE scores for the low-pyroptosis score group were significantly lower indicating patients with low pyroptosis scores tended to get effective response to immunotherapy (Figure 5F). The prognostic values of the pyroptosis score were confirmed in GSE78220 by Kaplan–Meier survival analysis (Figure 5G). Furthermore, patients with low pyroptosis scores were more likely to respond to anti-PD1 immunotherapy which was consistent with the above results (Figure 5H).

### Identification of the Correlation Between Pyroptosis Score and Clinicopathological Features

**FIGURE 6:**
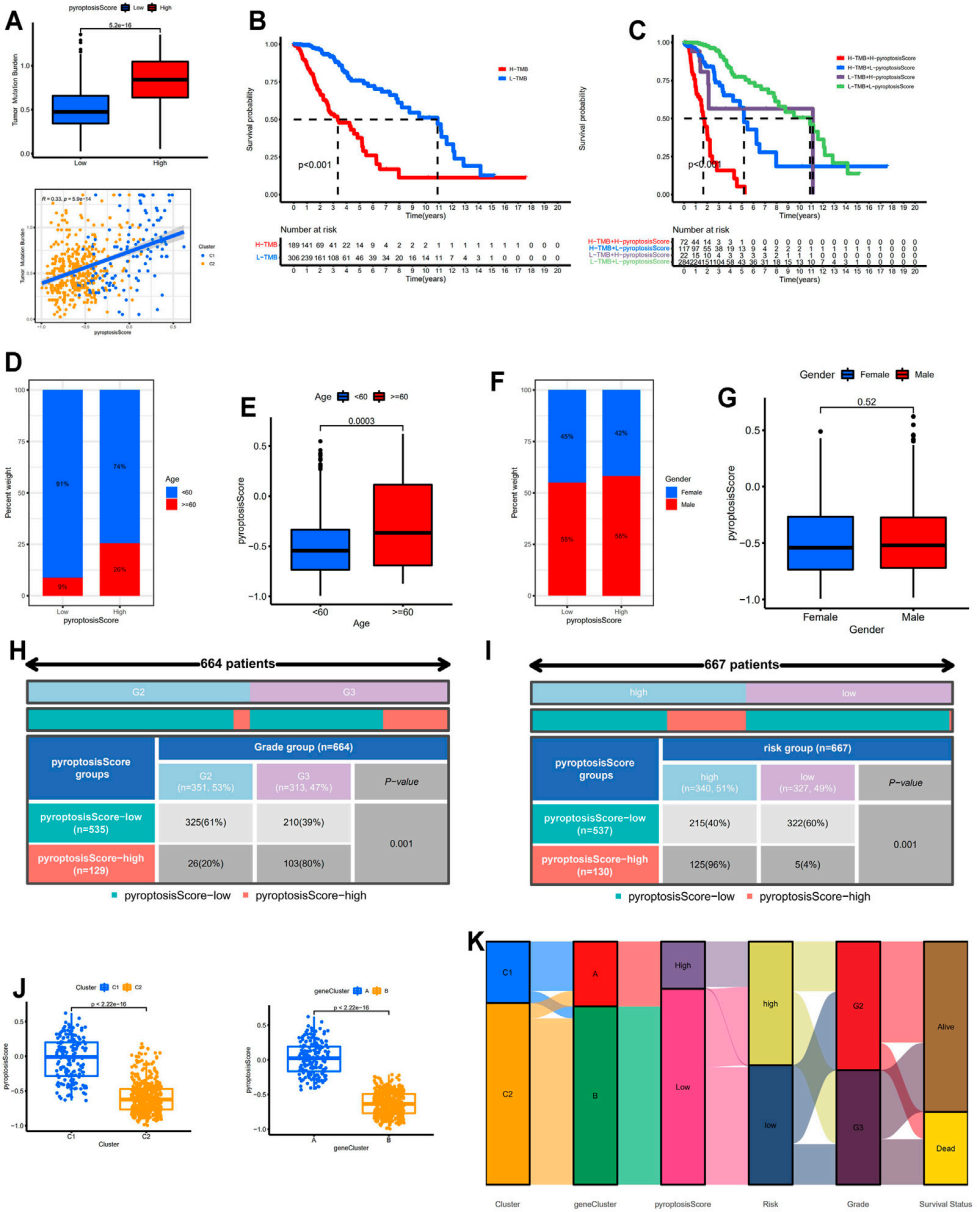
Correlation between pyroptosis score and clinicopathological features. **(A)** Identification of the correlation between pyroptosis score and TMB. **(B)** Kaplan–Meier survival analysis between the low- and high-TMB score groups. **(C)** Kaplan–Meier survival analysis for patients with different pyroptosis and TMB scores. **(D,E)** Correlation between pyroptosis score and age. **(F,G)** Correlation between pyroptosis score and gender. **(H)** Correlation between pyroptosis score and grade. **(I)** Correlation between pyroptosis score and risk group. **(J)** Comparisons of pyroptosis score between pyroptosis-related molecular patterns and gene clusters. **(K)** Alluvial diagram of pyroptosis patterns, gene clusters, pyroptosis score group, risk group, grade, and survival status. TMB: tumor mutation burden.

TMB, which closely correlated with TIME and served as a potential biomarker for immunotherapy response, was investigated in this study. TMB scores in the high-pyroptosis score group were significantly higher than those in the low-pyroptosis score group and the TMB score positively correlated with the pyroptosis score (Figure 6A). Patients with low TMB scores tended to get higher overall survival (*p* < 0.001; Figure 6B). The overall survival for patients with low pyroptosis scores and high TMB scores was significantly higher compared with patients with high pyroptosis scores and low TMB scores confirming that the pyroptosis score may be a robust and independent predictor for prognosis in LGGs (Figure 6C). Patients with low pyroptosis scores tended to be younger than those with high pyroptosis scores (Figures 6D,E). There was no correlation between pyroptosis score and gender (Figures 6F,G). LGG samples with high pyroptosis scores tended to present higher histological grade and higher risk score (*p* < 0.001; Figures 6H,I). The pyroptosis scores for patients in C1 were significantly higher than those in C2 and the pyroptosis scores for patients in gene cluster A were significantly higher compared with gene cluster B (Figure 6J). Alluvial diagram displayed the distribution of the LGG patients across pyroptosis patterns, gene clusters, pyroptosis score group, risk group, grade, and survival status (Figure 6K).

### Validation of Differential Expression of the Selected Genes in the Prognostic Model at Protein Level

**FIGURE 7:**
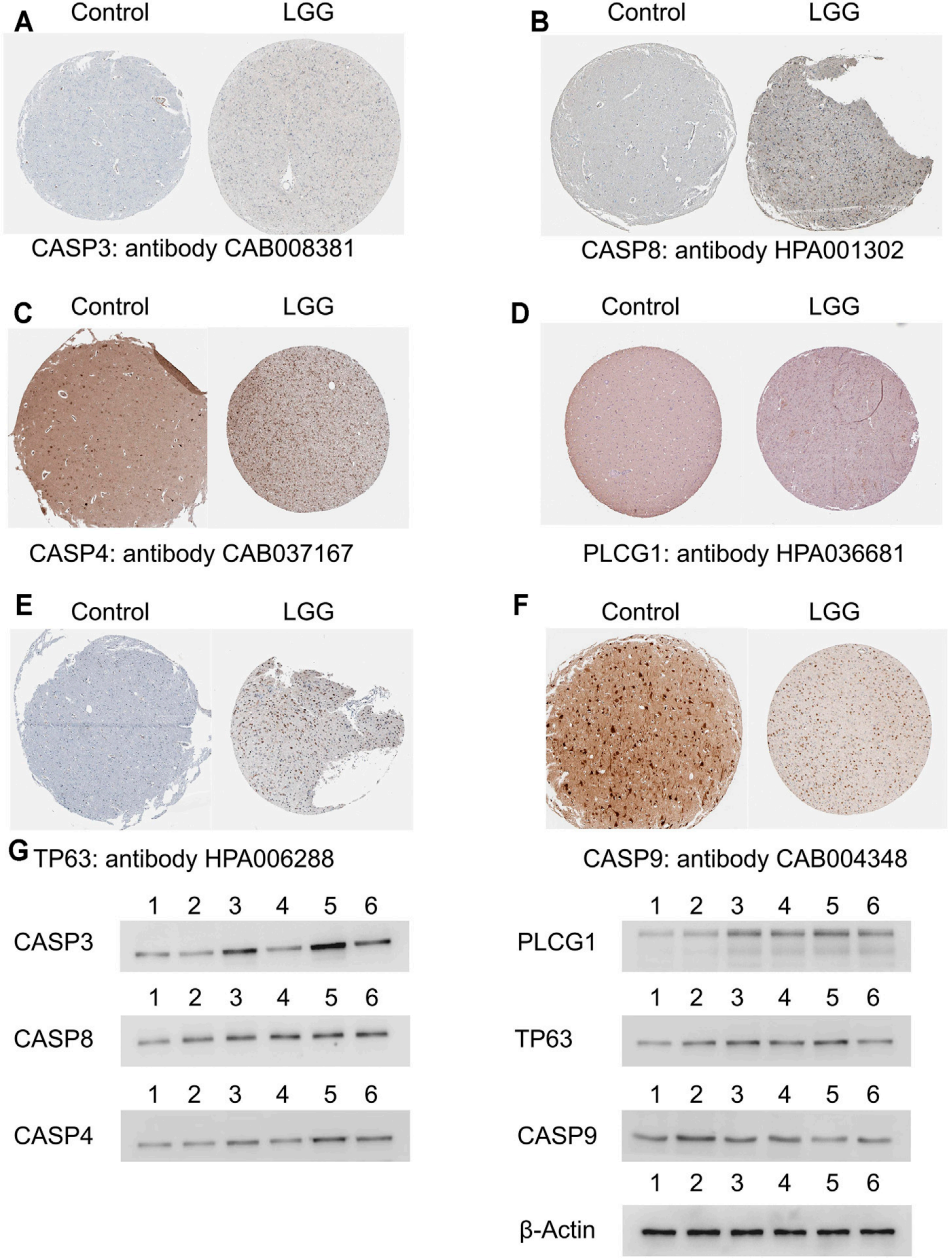
Validation of the selected genes in the prognostic model at the protein level. **(A–F)** Identification of the six selected genes in the prognostic model in immunohistochemistry staining. **(G)** Identification of the six selected genes in Western blotting in which lane 1 and 2 represented normal brain tissues, lane 3 represented the center tissues of glioma in grade II, lane 4 represented the margin tissues of glioma in grade II, lane 5 represented the center tissues of glioma in grade III, and lane 6 represented the margin tissues of glioma in grade III. LGG, low-grade glioma.

Six selected genes in the prognostic model including *CASP3*, *CASP8*, *CASP4*, *PLCG1*, *TP63*, and *CASP9* were scanned on The Human Protein Atlas website. We found that these genes were widely expressed in LGG tissues except *CASP9* which acted as favorable genes and were lowly expressed in LGG tissues (Figures 7A–F). In addition, Western blotting further verified the above results in which the expression levels of the unfavorable genes were relatively lower in normal brain tissues and the margin tissues of LGGs while the expression levels were extremely higher in the center tissues of LGGs (Figure 7G).

## Discussion

The workflow for this study was depicted in Supplementary Figure S3. Based on the expression profiles of 52 PRGs, LGG samples from TCGA and CGGA database were classified into two pyroptosis related molecular patterns with distinct prognosis and TIME. Besides, the prognostic model was constructed by using the LASSO-COX method to confirm the prognostic values of PRGs. Nomogram combing risk score and multiple clinicopathological characteristics were established for clinical practice. Furthermore, the pyroptosis score for each LGG sample was calculated through the GSVA method with appealing implications in predicting the prognosis and immune therapy in LGGs. The predictive performance of the pyroptosis score was subsequently validated in external data sets from CGGA and GEO database, respectively. Molecular biological experiments such as Western blotting analysis were employed to confirm the expression of the selected genes in the prognostic model at the protein level.

As depicted in the alluvial diagram, almost all the LGG samples in pyroptosis pattern C2 were stratified into gene cluster B and the latter one was completely defined as the low-pyroptosis score group. Moreover, LGG patients in C1 and gene cluster A with worse prognosis had significantly higher pyroptosis scores. Based on the findings in our study, we came to several conclusions: (1) the expression levels of most PRGs were highly expressed in pyroptosis pattern C1, gene cluster A, and high-pyroptosis score group, suggesting that pyroptosis was extensively activated; (2) to some extent, the pyroptosis score can serve as an indicator to distinguish pyroptosis-related molecular patterns and gene clusters for individuals; and (3) a high pyroptosis score indicated poor prognosis and immune checkpoint blockage therapeutic response while more immune cells infiltrated in the TIME of LGGs with high pyroptosis scores.

With the determination of gasdermins (GSDMs) protein family and inflammasomes, pyroptosis is emerging as an attractive target in cancer research for its indispensable effect on TIME and antitumor immunity (Zhang et al., 2020). To date, there are rare studies focusing on the topic of pyroptosis in gliomas and no pyroptosis related gene signature has been determined which were proved to have an important role in discriminating the prognosis and immunotherapy response in glioma patients in our study. Although pyroptosis was involved in various cancers, the specific roles for it can be complicated (Zhang et al., 2021). The tumor-suppressive effect of pyroptosis is proved in colorectal cancer, liver cancer, and skin cancer (Zaki et al., 2010; Ellis et al., 2011; Ma et al., 2016), but a double-edged effect is demonstrated in breast cancer (Chen et al., 2012). We cannot directly determine the effect of pyroptosis on the prognosis of patients based on the expression patterns of pyroptosis executors or regulators such as GSDMs alone. Thus, through scanning literatures, we screened out 52 PRGs involved in all the known pathways associated with pyroptosis to identify pyroptosis-related molecular patterns, followed which DEGs between two pyroptosis patterns were determined as pyroptosis-related gene signatures. Gasdermins (GSDMs) represent a recently identified protein family which is considered as the mediator and executor of pyroptosis. *GSDMD,* which was extensively investigated in various cancers, was involved in the unfavorable pyroptosis gene set in this study. The high expression of *GSDMD* in pyroptosis pattern C1 and gene cluster A accounted for the activation of pyroptosis in LGGs to some extent. A recent study indicated that pyroptosis can be triggered through TNF-mediated death receptor signaling pathway (Hou et al., 2020). Consistently, the function enrichment analysis in our study revealed that tumor necrosis factor activated receptor activity, Toll-like receptor signaling pathway, and natural killer cell mediated cytotoxicity pathway were significantly enriched in C1, which probably suggested the activation of pyroptosis in C1. Even though high expression of PRGs indicated poor prognosis in LGGs in our study, the specific effects of pyroptosis on the prognosis of patients remained unclear.

Our study provided strong evidence for the clinical management of LGG patients and made a step in the identification of pyroptosis in LGGs at the transcriptional and protein levels. First, given that the pyroptosis score was calculated for each sample, it can be an indicator for the heterogeneity of tumors and contribute to the development of personalized medicine. Second, the pyroptosis score took favorable and unfavorable gene signatures into account and closely correlated with prognosis which differed with other prognostic models mainly consisting of tumor promoting genes. Third, except for the prognostic values, the pyroptosis score significantly correlated with multiple clinicopathological features such as TMB and histological grade. Moreover, it can serve as a predictor for immune checkpoint blockage therapeutic response. Finally, the pyroptosis scoring scheme was verified in melanomas implying that it may also apply to other types of tumors.

Although multi-level and multi-database research were involved in the validations, there are still some limitations for our research. Tumor heterogeneity was not fully investigated even though the personalized differences were taken into consideration in this study. As shown in the Western blotting, the expression levels of the six selected genes involved in the prognostic model differed between tissues from the central area and margin area of LGGs. Single cell analysis focusing on the alterations of PRGs may be required in future research to explore the heterogeneity of LGGs. Previous studies revealed the tumor suppressive effect of pyroptosis in cancers (Saeki et al., 2009; Grivennikov et al., 2010; Wang et al., 2018), while in this study, we found that LGG patients with high pyroptosis scores exhibited poor prognosis. The activation of pyroptosis in which PRGs were highly expressed may act as an indicator or result of tumor progression. As for LGGs with high aggressiveness and proliferation, pyroptosis may raise as a general innate immune effector mechanism to sustain the balance of TIME, therefore, induction of pyroptosis by targeted drugs may augment the antitumor effect. Additionally, this study concentrated on the alterations of pyroptosis related genes or proteins and more *in vitro* and *in vivo* experimental evidence at the resolution of cells might be needed for extensive research of pyroptosis in the future. Moreover, pyroptosis has been recently defined as a type of PANoptosis which represents an inflammatory programmed cell death pathway. PANoptosis can be regulated by the PANoptosome complex that shares common features with pyroptosis, apoptosis, and/or necroptosis but that cannot be accounted for by any of these three pathways alone (Wang and Kanneganti, 2021). Although our study provided a comprehensive analysis of pyroptosis related genes in LGGs and shed light on the investigation of PANoptosis, an in-depth analysis focusing on the exploration of PANoptosis would draw more attention in the future.

In conclusion, we classified LGG patients into subgroups with different pyroptosis related molecular patterns. Pyroptosis scoring scheme was developed to further characterize pyroptosis in LGGs with implications in discriminating the prognosis and immunotherapy responses. LGG patients with lower pyroptosis scores usually got better prognosis and tended to benefit from immune checkpoint blockage therapy.

## Data Availability

Publicly available datasets were analyzed in this study. This data can be found here: The RNA-seq data consisting of LGGs samples from TCGA database (https://portal.gdc.cancer.gov/), two data sets (DataSet ID: mRNA-array_301, mRNAseq_325) from CGGA database (http://cgga.org.cn/index.jsp), four data sets (GSE4271, GSE4412, GSE43378, GSE84010) obtained from the Gene Expression Omnibus (GEO, https://www.ncbi.nlm.nih.gov/geo/) database.
