# Supplementary material for: Comprehensive Characterization of Pyroptosis Patterns with Implications in Prognosis and Immunotherapy in Low-Grade Gliomas
Source: Front Genet. 2022 Feb 7;12:763807. doi: 10.3389/fgene.2021.763807 (PMC8859270; doi:10.3389/fgene.2021.763807)
Supplement: Supplementary file 1 [file Table1.DOCX]

**Supplementary table 1. Clinical features of patients with low-grade gliomas in CGGA database (DataSet ID: mRNAseq_325)**

| Covariates |  | Total | High-pyroptosisScore | Low-pyroptosisScore |
| --- | --- | --- | --- | --- |
| Gender | Female | 66(38.37%) | 33(39.29%) | 33(37.5%) |
|  | Male | 106(61.63%) | 51(60.71%) | 55(62.5%) |
| Age | <60 | 160(93.02%) | 73(86.9%) | 87(98.86%) |
|  | >=60 | 12(6.98%) | 11(13.1%) | 1(1.14%) |
| Grade | G2 | 98(56.98%) | 29(34.52%) | 69(78.41%) |
|  | G3 | 74(43.02%) | 55(65.48%) | 19(21.59%) |
| Type | Primary | 137(79.65%) | 58(69.05%) | 79(89.77%) |
|  | Recurrent | 35(20.35%) | 26(30.95%) | 9(10.23%) |
| Radio Status | treated | 139(80.81%) | 63(75%) | 76(86.36%) |
|  | untreated | 29(16.86%) | 18(21.43%) | 11(12.5%) |
|  | unknow | 4(2.33%) | 3(3.57%) | 1(1.14%) |
| Chemo Status  (TMZ) | treated | 89(51.74%) | 49(58.33%) | 40(45.45%) |
|  | untreated | 75(43.6%) | 32(38.1%) | 43(48.86%) |
|  | unknow | 8(4.65%) | 3(3.57%) | 5(5.68%) |
| IDH Status | Mutant | 127(73.84%) | 50(59.52%) | 77(87.5%) |
|  | Wildtype | 44(25.58%) | 34(40.48%) | 10(11.36%) |
|  | unknow | 1(0.58%) | 0(0%) | 1(1.14%) |
| 1p19q  codeletion  Status | Codel | 55(31.98%) | 7(8.33%) | 48(54.55%) |
|  | Non-codel | 115(66.86%) | 77(91.67%) | 38(43.18%) |
|  | unknow | 2(1.16%) | 0(0%) | 2(2.27%) |
| MGMTp  methylation  Status | methylated | 85(49.42%) | 36(42.86%) | 49(55.68%) |
|  | un-methylated | 71(41.28%) | 37(44.05%) | 34(38.64%) |
|  | unknow | 16(9.3%) | 11(13.1%) | 5(5.68%) |
