# Supplementary figures and images for: Comprehensive Characterization of Pyroptosis Patterns with Implications in Prognosis and Immunotherapy in Low-Grade Gliomas
Source: Front Genet. 2022 Feb 7;12:763807. doi: 10.3389/fgene.2021.763807 (PMC8859270; doi:10.3389/fgene.2021.763807)

A

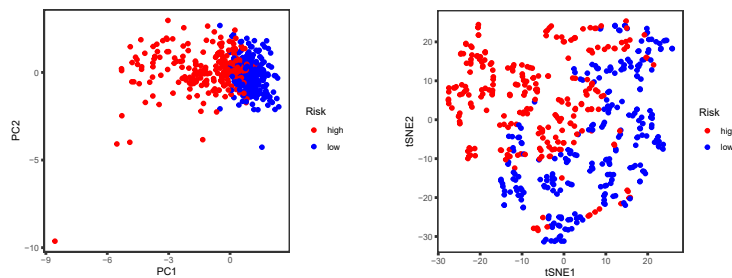

B

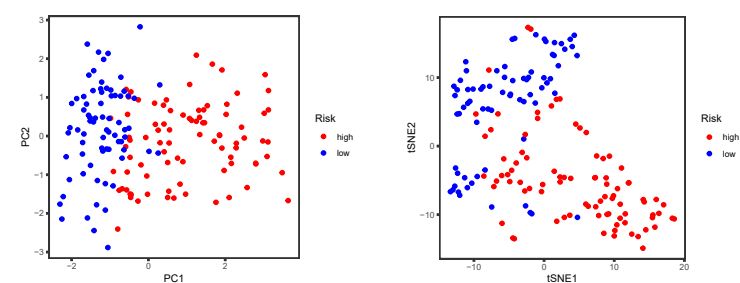

C

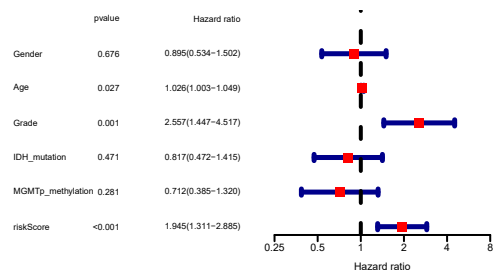

D

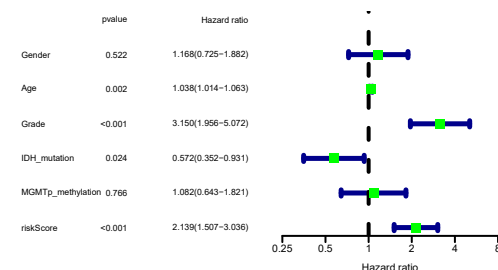

E

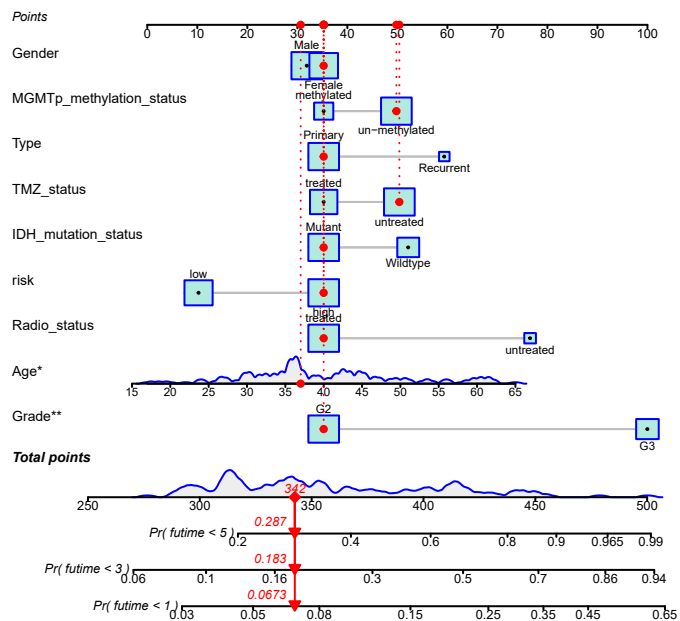

F

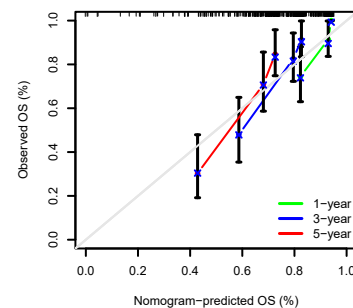

G

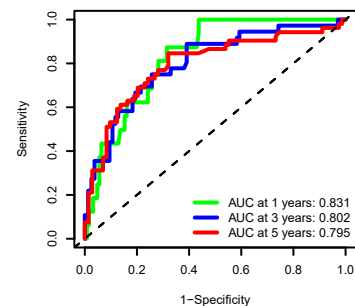

Supplement: Supplementary file 2 [file Image2.PDF]

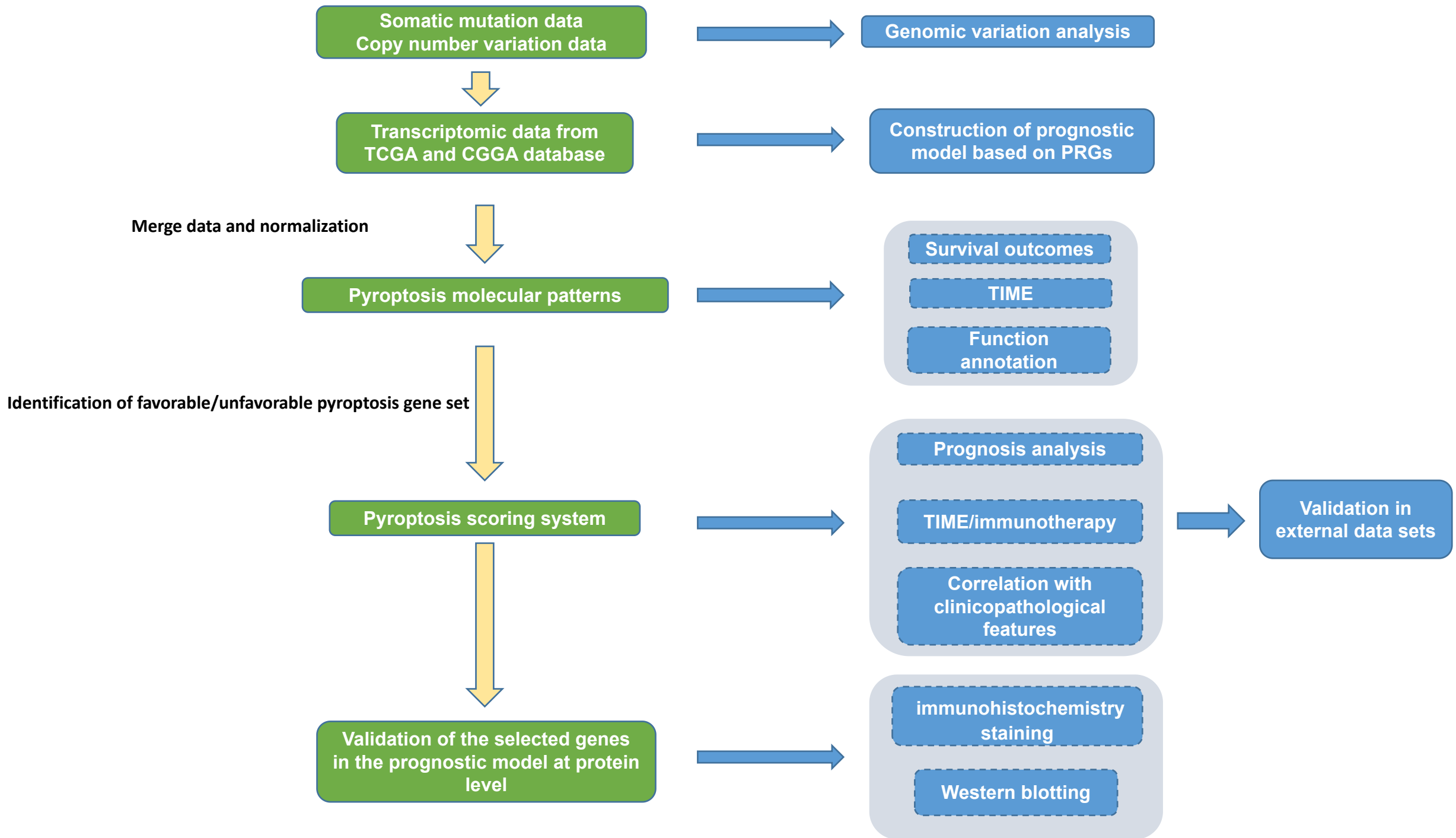

Supplement: Supplementary file 3 [file Image3.PDF]
