# Supplementary material for: Comprehensive Characterization of Pyroptosis Patterns with Implications in Prognosis and Immunotherapy in Low-Grade Gliomas
Source: Front Genet. 2022 Feb 7;12:763807. doi: 10.3389/fgene.2021.763807 (PMC8859270; doi:10.3389/fgene.2021.763807)
Supplement: Supplementary file 4 [file Table2.DOCX]

**Supplementary table 2. Clinical features of patients with low-grade gliomas in GEO database (GSE4271,** **GSE4412, GSE43378, GSE84010)**

| Covariates |  | Total | High-pyroptosisScore | Low-pyroptosisScore |
| --- | --- | --- | --- | --- |
| Gender | Female | 51(46.36%) | 33(45.83%) | 18(47.37%) |
|  | Male | 59(53.64%) | 39(54.17%) | 20(52.63%) |
| Age | <60 | 103(93.64%) | 67(93.06%) | 36(94.74%) |
|  | >=60 | 7(6.36%) | 5(6.94%) | 2(5.26%) |
| Grade | G2 | 5(4.55%) | 0(0%) | 5(13.16%) |
|  | G3 | 105(95.45%) | 72(100%) | 33(86.84%) |
