# Supplementary material for: Comprehensive Characterization of Pyroptosis Patterns with Implications in Prognosis and Immunotherapy in Low-Grade Gliomas
Source: Front Genet. 2022 Feb 7;12:763807. doi: 10.3389/fgene.2021.763807 (PMC8859270; doi:10.3389/fgene.2021.763807)
Supplement: Supplementary file 5 [file Image1.PDF]

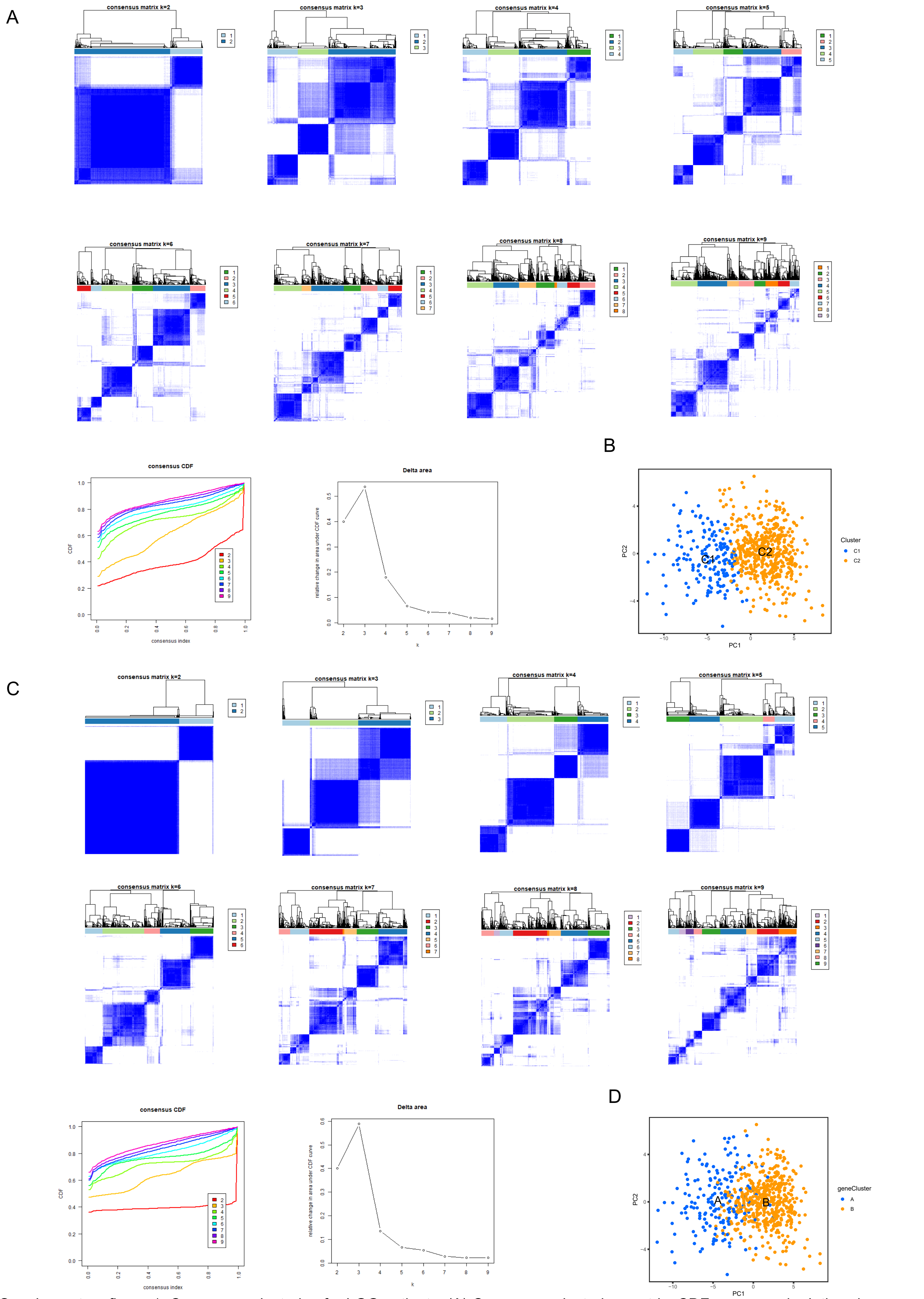

Supplementary figure 1. Consensus clustering for LGG patients. (A) Consensus clustering matrix, CDF curves and relative change in the area under the CDF curve (k = 2-9) for consensus clustering analysis based on PRGs in LGGs. (B) PCA of the expression profiles of PRGs from LGG samples confirmed the two pyroptosis related molecular patterns, C1(blue) and C2 (yellow). (C, D) Similar results of consensus clustering analysis based on DEGs between two pyroptosis related molecular patterns in LGGs. LGG, low-grade glioma; CDF, cumulative distribution function; PRG, pyroptosis related genes; PCA, principal component analysis; DEG, differentially expressed gene.
